# Room-Temperature Synthesis of Pullulan-Based Hydrogels for Controlled Delivery of Microbial Fertilizers

**DOI:** 10.3390/polym17243323

**Published:** 2025-12-16

**Authors:** Tamara Erceg, Ivana Mitrović, Vesna Teofilović, Darko Micić, Sanja Ostojić

**Affiliations:** 1 Faculty of Technology Novi Sad, University of Novi Sad, Bulevar cara Lazara 1, 21000 Novi Sad, Serbia; tadi@uns.ac.rs (I.M.); vesnateofilovic@uns.ac.rs (V.T.); 2 Institute of General and Physical Chemistry, University of Belgrade, Studentski trg 12/V, 11158 Beograd, Serbia; micic83@gmail.com (D.M.); ostojicsanja404@gmail.com (S.O.)

**Keywords:** metharylated pullulan, free-radical polymerization, microbial fertilizer, microbial suspension-loaded hydrogels

## Abstract

This study presents an energy-efficient, room-temperature synthesis and characterization of methacrylated pullulan (Pull-MA) hydrogel developed for controlled nutrient delivery in agricultural applications. Fourier Transform Infrared Spectroscopy (FTIR) and Differential Scanning Calorimetry (DSC) analyses confirmed the successful functionalization of pullulan with methacrylate groups, accompanied by a decrease in thermal transition temperatures, indicative of increased polymer chain mobility. The synthesized Pull-MA hydrogel exhibited a high swelling capacity, reaching an equilibrium swelling ratio of 1068% within 5 h, demonstrating its suitability as a carrier matrix. The room-temperature synthesis approach enabled the in situ incorporation of microbial inoculant into the hydrogel network, preserving microbial viability and activity. SEM analysis performed under the different magnifications (1000, 2500, 5000, 10,000, 25,000×) has confirmed brittle nature of xerogels and increasing in structural irregularities with increasing in cultivation broth content.The biological performance of the fertilizer-loaded hydrogels was evaluated through seed germination assays using maize and pepper as model crops. The optimized formulation, T2 (Pull-MA: cultivation broth 1:5 *w*/*w*), significantly improved germination efficiency, as evidenced by increased relative seed germination (RSG), root growth rate (RRG), and germination index (GI) compared to both the control and the low-fertilizer formulation (T1, 1:2.5 *w*/*w*). These findings highlight the potential of Pull-MA hydrogels as bioactive seed-coating materials that enhance early seedling development through controlled nutrient release. The results lay a solid foundation for further optimization and future application of this system under real field conditions.

## 1. Introduction

The escalating global population and the imperative for sustainable agricultural practices necessitate innovative approaches to enhance food production while minimizing environmental impact. Conventional agriculture, heavily reliant on synthetic chemical fertilizers, has demonstrably contributed to soil degradation, water pollution, and greenhouse gas emissions [1,2]. In response, the adoption of microbiological fertilizers, also known as biofertilizers, has emerged as a promising and eco-friendly alternative. Bio-fertilizers, which comprise beneficial microorganisms (bacteria and fungi), enhance plant growth by improving nutrient availability, promoting soil health, and increasing plant resilience against environmental stressors [3,4]. Various authors have investigated different bacterial strains for their ability to promote plant growth, including *Rhizobium* [5], *Bacillus* [6], *Pseudomonas* [7], *Azobacter* [8], and *Azospirilum* [9]. Among fungal species, members of the genera *Rhizophagus* [10], *Suillus* [11], *Aspergillus* [12], *Penicillium* [13], and *Trichoderma* [14] have demonstrated notable potential as biofertilizers due to their ability to enhance nutrient availability, stimulate plant growth, and improve soil health.

However, the efficacy of bio-fertilizers is often compromised by the transient viability of the microorganisms in the dynamic soil environment, coupled with their susceptibility to rapid leaching and inconsistent distribution following application [15,16]. Consequently, there is an urgent need for advanced delivery systems that can protect beneficial microbes and facilitate their sustained, controlled release into the rhizosphere, thereby maximizing their biological activity and prolonged efficacy. Within this rapidly developing field, biopolymers such as proteins and polysaccharides have gained significant attention as encapsulation matrices due to their natural abundance, biodegradability, biocompatibility, and non-toxicity [17]. Polysaccharides, including alginate, chitosan, starch, maltodextrin, and gum arabic, as well as proteins such as gelatin and whey protein, have been employed as carrier materials for the encapsulation of beneficial microorganisms using various techniques such as spray drying, extrusion, and emulsification.

The primary role of these carriers is to encapsulate microbial strains (e.g., *Pseudomonas* or *Bacillus* spp.) [17] or nutrients, providing essential physical and chemical protection against environmental stresses such as desiccation, UV radiation, and abrupt pH fluctuations [18]. This protective barrier markedly enhances the viability and shelf-life of the encapsulated microbial agents. In addition to microencapsulation, extrusion, and emulsification techniques, hydrogel formation has emerged as a powerful strategy for designing advanced delivery systems. By engineering hydrogels, it is possible to precisely modulate the biopolymer matrix’s degradation kinetics and align the release profile of active agents with the specific physiological needs and growth stages of plants. Hydrogels are three-dimensional polymer networks that can absorb and retain large amounts of water. They are increasingly recognized as highly efficient matrices for the encapsulation and controlled release of sensitive biological agents. Their inherent biocompatibility, tunable swelling properties, and porous network structure make them particularly suitable for applications in both biomedical and agricultural sectors. Compared to other encapsulation techniques, polysaccharide-based hydrogels offer additional advantages, including lower production cost, simpler processing steps, and the use of mild conditions that help preserve microbial viability and bioactivity. These features make hydrogels a highly attractive platform for the development of next-generation biofertilizer delivery systems [19,20].

Pullulan hydrogels are widely recognized as promising biopolymer carriers, primarily due to pullulan’s excellent biocompatibility, non-toxicity, and existing approval for food and biomedical applications [21,22]. Through chemical modifications—such as oxidation (to form dialdehyde pullulan) or blending with other polymers (e.g., starch)—pullulan forms stable, three-dimensional hydrogel networks allowing for the precise tailoring of hydrogel properties such as mechanical strength, swelling capacity, and degradation kinetics to meet specific application requirements [23]. These networks act as microcapsules that protect the incorporated nutrients or microorganisms from unfavorable soil conditions while controlling their diffusion, allowing for the precise tailoring of hydrogel properties such as mechanical strength, swelling capacity, and degradation kinetics to meet specific application requirements [24]. Furthermore, pullulan is fully biodegradable by soil microorganisms, ensuring that the hydrogel matrix ultimately breaks down into innocuous byproducts, aligning perfectly with principles of sustainable agriculture [25]. A quantitative encapsulation of microbial suspensions within a hydrogel matrix can be effectively achieved only via in situ incorporation, ensuring maximal retention of viable cells and bioactive metabolites. Therefore, hydrogel synthesis must be conducted at room temperature to avoid thermal degradation of microbial cells and nutrient components.

In the available literature, pullulan-based hydrogels intended for microbial suspension incorporation are predominantly obtained through Schiff base formation or other chemical crosslinking strategies that avoid high temperatures. However, no reports have been found on the preparation of pullulan-based hydrogels via free-radical polymerization, particularly under ambient conditions. In this study, *Trichoderma harzianum* cultivation broth, used as a microbiological fertilizer, was incorporated in situ into a pullulan-based hydrogel network synthesized by free-radical polymerization at room temperature. The work explores the design, synthesis, characterization, and potential application of these hydrogels, specifically engineered to enable the controlled release of microbial fertilizers. Ultimately, this research aims to advance sustainable agricultural practices by developing a robust, low-energy, and environmentally benign delivery system for microbiological fertilizers, potentially enhancing both fertilizer efficiency and soil microbiome health.

## 2. Materials and Methods

### 2.1. Materials

Pullulan (Mw~574,570 g/mol) was procured from Avena Lab (Vršac, Srbija). Glycidyl methacrylate (GMA), *N,N*′-methylenebisacrylamide (MBAM), and *N,N,N′*, *N′*-tetramethylethylenediamine (TEMED) were procured from Sigma Aldrich (St. Louis, MO, USA). Dimethyl sulfoxide (DMSO) and potassium persulfate (PPS) were supplied by CENTROHEM (Stara Pazova, Serbia). A catalyst, 4-dimethylaminopyridine (DMAP), was provided by Thermo Fisher Scientific Inc. (Waltham, MA, USA). The seeds of *Capsicum annuum* L. “Somborka” were purchased from the producer SemeSemena (Belgrade, Serbia). Untreated seeds of commercial maize hybrid from Serbian market were used in this study.Phosphate-buffered saline (PBS) (pH 7.4) was supplied from Thermo Fisher Scientific (Waltham, MA, USA).

### 2.2. Preparation of Biofertilizer

*Trichoderma harzianum* cultivation broth was prepared on an optimized medium according to the instructions described by Mitrović et al. [26]. Briefly, an optimized medium with the following composition was used for the cultivation of *T. harzianum*: dextrose (10 g/L), soybean flour (6.87 g/L), K_2_HPO_4_ (1.51 g/L), KCl (0.5 g/L), and MgSO_4_ × 7H_2_O (0.5 g/L). Cultivation was performed in 300 mL Erlenmeyer flasks with 100 mL of medium. Before cultivation, *T. harzianum* was grown on PDA (Potato Dextrose Agar) for 10 days, and then a piece of mycelium from the edge of the Petri plate was taken for the preparation of liquid inoculum. Liquid inoculum was prepared on PDB (Potato Dextrose Broth) medium for 4 days on a rotary shaker at a temperature of 27 ± 1 °C and 170 rpm. A 10% (*v*/*v*) of prepared liquid inoculum was used for the experiment. After 96 h of cultivation on a rotary shaker at 27 ± 1 °C, the cultivation broth was filtered through a double layer of sterile cheesecloth, and the liquid part was used for hydrogel preparation.The concentration of multiplied T. harzianum cells before hydrogel preparation was examined using an automated cell counter (Countess, ThermoFisher, Waltham, MA, USA) [27]. The production microorganism is kept in the Collection of Microbial Cultures of the Faculty of Technology, Novi Sad, Serbia.

### 2.3. Preparation of Neat and Loaded Hydrogel

**Figure 1:**
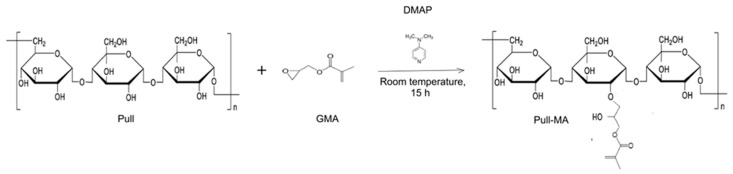
Scheme of the Pull modification by GMA.

Hydrogels were synthesized through a two-step process, with the first step involving the chemical modification of pullulan. The pullulan solution was prepared in dimethylsulfoxide (DMSO) in a mass ratio of 1:4. Homogenization was carried out at a temperature of 55–60 °C until the complete dissolution of pullulan, after which the solution was cooled to room temperature. Then, the catalyst DMAP was added in a mass ratio of biopolymer/catalyst = 10:1. After the complete dissolution of the catalyst, GMA was added in a mass ratio of biopolymer/GMA = 1:1.6. The functionalization reaction was carried out for 15 h with continuous stirring at room temperature. Modified pullulan-pullulan methacrylate (Pull-MA) was precipitated first with acetone, then with acetone and ethanol, and dried at 50 °C until a constant mass. The reaction of pullulan modification using GMA is presented in Figure 1. In the second step, the hydrogel was prepared by dissolving Pull-MA in distilled water (1:6 mass ratio), with intensive homogenization at 50 °C for 60 min.

**Figure 2:**
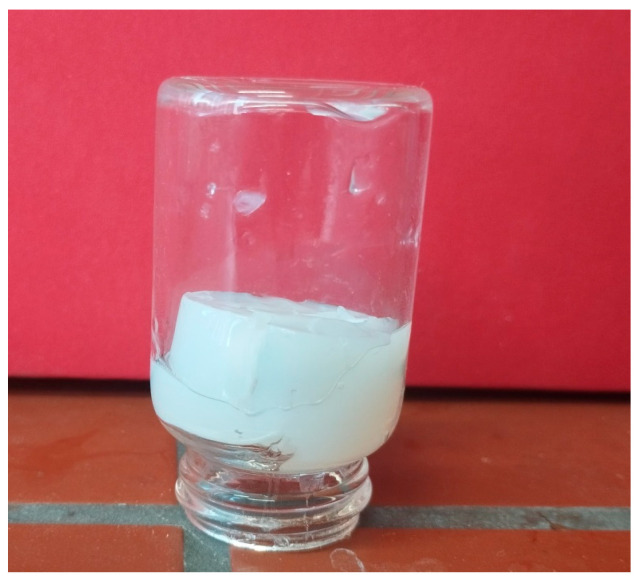
Visual appearance of Pull-MA hydrogel.

After that, the solution was cooled to room temperature, and with continued stirring, 1 mL of an aqueous solution of MBAM (0.04 g/mL), 0.5 mL of a solution of TEMED (0.03 g/mL), and 1 mL of an initiator solution of PPS (0.04 g/mL) were added. Within 2 min at room temperature, a gel was formed (Figure 2).

Microbiological suspension-loaded hydrogels were prepared by in situ incorporation of *T. harzianum* cultivation broth (3 × 10^8^ CFU/mL),. Microbiologically active hydrogels were obtained by in situ incorporation of the *T. harzianum* cultivation broth into the hydrogel matrix. One control sample (without cultivation broth) and three hydrogel samples containing different amounts of cultivation broth were prepared with the following compositions (weight ratios):

T1. Pull-MA: cultivation broth = 1: 7.5

T2. Pull-MA: cultivation broth = 1:5

T3. Pull-MA: cultivation broth = 1:2.5.

These specific ratios were selected based on preliminary optimization experiments of the modified biopolymer–cultivation broth system. At higher broth concentrations, free-radical polymerization was inhibited, preventing effective crosslinking and thus hindering gel point achievement. Conversely, lower broth concentrations resulted in a reduced biological effect on germination. Therefore, these intermediate ratios, applied with regular spacing, were chosen as a compromise between polymerization feasibility and biological efficiency.

### 2.4. Characterization Techniques

The chemical structures of neat pullulan, pullulan-MA, and the obtained hydrogel were characterized using Fourier-transform infrared spectroscopy (FTIR, Waltham, MA, USA) in attenuated total reflectance (ATR) mode. Spectra were recorded with a resolution of 4 cm^−1^ over the wavenumber range of 4000–400 cm^−1^.

Differential scanning calorimetry (DSC) analysis was performed to confirm the modification of pullulan using a TA Instruments Q20 calorimeter (New Castle, DE, USA) under a nitrogen atmosphere (flow rate 50 mL/min). The instrument was calibrated for temperature and cell constant using indium reference samples, while specific heat capacity (*C_p_*) calibration was carried out with a sapphire standard, both supplied by TA Instruments. Each sample was heated from 25 to 250 °C at a rate of 10 °C/min in one cycle. The melting temperature (*T_m_*) was determined from the endothermic peak maximum, while the glass transition temperature (*T_g_*) was defined as the midpoint of the heat capacity change (*∆C_p_*). The standard uncertainty of temperature measurements was u (T) = 0.5 °C.

Scanning electron microscopy (SEM) imaging of the neat and microorganism-loaded xerogels was performed using a JEOL JSM-6610LV microscope (Tokyo, Japan) equipped with an Oxford Instruments X-Max SDD EDS detector (Abingdon, UK). The analyses were carried out at an accelerating voltage of 20 kV. Samples were coated with gold and investigated under the different magnifications (1000; 2500; 5000; 10,000; 25,000×).

The swelling behavior of the neat hydrogel in the granulated state was investigated at room temperature (23 ± 2 °C) at neutral pH, using the tea bag method. Before the experiment, the xerogel was precisely weighed and immersed in distilled water at room temperature. After predefined time intervals, the samples were taken out of the water, carefully wiped off the surface, and weighed again. The equilibrium swelling ratio (ESR) was determined according to the following equation:

(1) $$ESR=\frac{m_{t}-m_{0}}{m_{0}}100$$

where m_0_ is the mass of the dry sample (in grams) and m_t_ is the mass of the swollen hydrogel (in grams) after a certain time interval (measured every 1 h for a total of 5 h). Measurement was carried out in triplicate and average values were determined.

To investigate the efficiency of crosslinking, the gel fraction (GF) was determined, giving information about the amount of network structure insoluble in water. The procedure was carried out by placing 0.2 g of dried, granulated xerogel in 20 mL of water at 37 °C, leaving it to swell for 5 h until reaching ESR. After 5 h, swollen hydrogel granules were separated using a strainer and dried until constant weight. The GF was calculated as the ratio of the remaining dry mass of the gel network (m_g_) to the initial dry mass of the sample (m_d_), expressed as a percentage:

(2) $$GF=\frac{m_{g}}{m_{d}}\cdot 100\%$$

Measurements were carried out in triplicate and average values were determined.

For the seed germination test, fifty maize (*Zea mays*) seeds and fifty pepper (*C. annuum*) seeds were used for each test (5 Petri plates × 10 seeds). Just before the experiment, the seeds were surface sterilized for 5 min with 1% sodium hypochlorite solution and then washed with sterile distilled water. Five tests were applied in the experiment: K1 w–control 1, sterile distilled water; K2g–control 2, neat hydrogel; T1, T2, and T3—hydrogels with the addition of *T. harzianum* as explained in Section 2.3. According to the slightly modified method of Ranucci et al. (2024) [28], two 85 mm diameter filter papers were added to polystyrene Petri plates with a diameter of 90 mm, on which 10 seeds were evenly distributed. Additionally, 0.1 g of the appropriate hydrogel was added to tests K2g, T1, T2, and T3 before adding 5 ml of water. After adding water, 10 seeds were placed in Petri plates, sealed with Parafilm, and incubated at a temperature of 25 ± 1 °C with a natural day-night cycle. In accordance with the ISTA (International Seed Testing Association) procedure, after 4 days for maize and after 7 days for peppers, germinated seeds were counted. The results of germinated seeds were statistically processed to determine the significance levels among the treatments.

Root length was measured after 7 days for maize and after 14 days for pepper (ISTA). RRG—relative radicle growth was determined using equation 3. Also, RSG—relative seed germination, SG—seed germination percentage, and GI—germination index were determined using the equations (4-6) explained in detail in the paper by Ranucci et al. (2024) [28].

(3) $$RRG=\frac{Average\;radicle\;length\;in\;test\;sample}{Average\;radicle\;length\;in\;water}$$

(4) $$RSG=\frac{Number\;of\;germinated\;seeds\;exposed\;to\;test\;hydrogels}{Number\;of\;germinated\;seeds\;exposed\;to\;water}$$

(5) $$SG\left(\%\right)=\frac{Number\;of\;germinated\;seeds}{Number\;of\;tested\;seeds}\cdot 100\%$$

(6) GI=RRG×RSG

Vival rate of *T. harzianum* encapsulated in Pull-MA hydrogel was evaluated using phosphate-buffered saline (PBS) according to Oberoi et al. (2021) [29] with appropriate modifications. Individual hydrogel discs of uniform size were placed into sterile tubes containing 10 mL of sterile PBS (pH 7.4) and incubated at 25 °C under gentle agitation of 50 rpm. At defined time points: 0, 1, 2, 4, 12, 24, 48, and 96 h, followed by 7, 14, and 28 days, aliquots (0.5 mL) were aseptically withdrawn from the incubation medium in three replicates for each defined time interval. The number of viable microorganisms was quantified by the colony-forming unit (CFU) method using serial dilutions and plating on PDA, followed by incubation at 28 °C for 48–72 h. The survival rate (%) was calculated using the equation:

(7) $$Survival\;rate\left(\%\right)=\left(\frac{Nt}{No}\right)\times 10$$

where Nt is the number of viable cells after a defined time t in PBS conditions, and N_0_ is the initial number of viable cells at the moment of hydrogel formation. All experiments were performed in triplicate, and the results were presented as mean values ± standard deviation.

### 2.5. Data Analysis

Results of the seed germination test were processed by one-way ANOVA using Software Statistica, version 14.0 (StatSoft Inc., Tulsa, OK, USA). Duncan’s multiple range test was used to test the significance of differences (*p* ≤ 0.05).

## 3. Results and Discussion

### 3.1. Results of Structural Analysis

**Figure 3:**
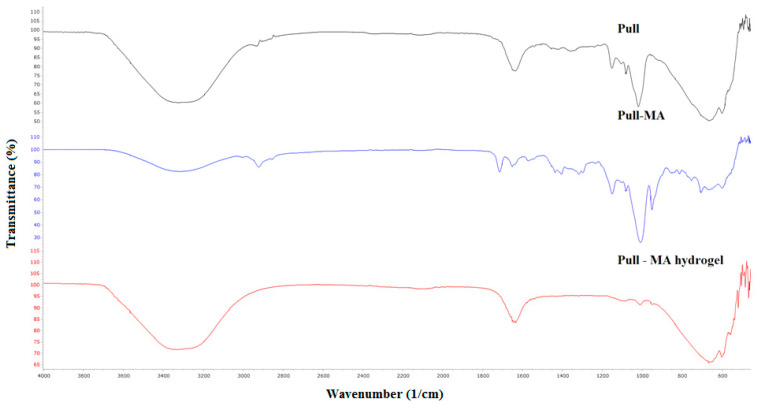
FTIR spectra of Pull, Pull-MA, and Pull-MA hydrogel.

FTIR spectra of neat pullulan, Pull-MA, and Pull hydrogel are presented in Figure 3. A broad band with the center at 3320 cm^−1^ (Pull), 3322 cm^−1^ (Pull-MA), and 3335 cm^−1^ (Pull gel) is attributed to the OH stretching vibrations of the Pull structure. Two peaks between 2800 and 2900 cm^−1^ correspond to the CH stretching vibrations. The peak that appears only in the FTIR spectrum of Pull-MA at 1715 cm^−1^ is attributed to the C=O bond, which confirms the success of the modification of Pull by GMA. In all three spectra, there is are absorption band at 1642—1632 cm^−1^ originating from the O—C—O bond in the polysaccharide skeleton. Two peaks between 1437 and 1361 cm^−1^ in the FTIR spectrum of neat Pull are attributed to CH_2_ and CH_3_ bending.

### 3.2. Results of DSC Analysis

**Figure 4:**
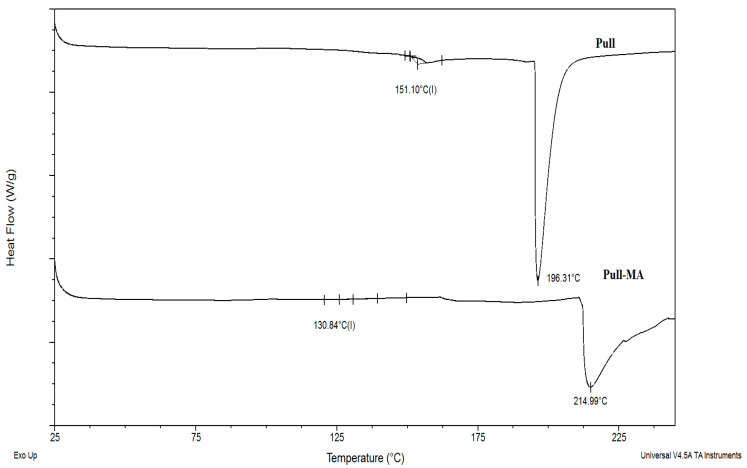
DSC thermograms for neat Pull and Pull-MA.

Differential scanning calorimetry (DSC) thermograms were recorded for neat Pull and Pull-MA to compare their thermal transitions (Figure 4). For neat pullulan, a glass transition temperature (*T_g_*) was observed at 151 °C. After the incorporation of methacrylate groups, the segmental mobility of the polymer chains increased, leading to a reduction in *T_g_* to 130 °C. As a generally amorphous biopolymer, pullulan does not exhibit a melting peak in its DSC curve [30]. The thermal degradation peak appeared at 196 °C for pullulan and shifted to 214 °C for Pull-MA, indicating enhanced thermal stability after methacrylation.

### 3.3. Results of SEM Analysis

**Figure 5:**
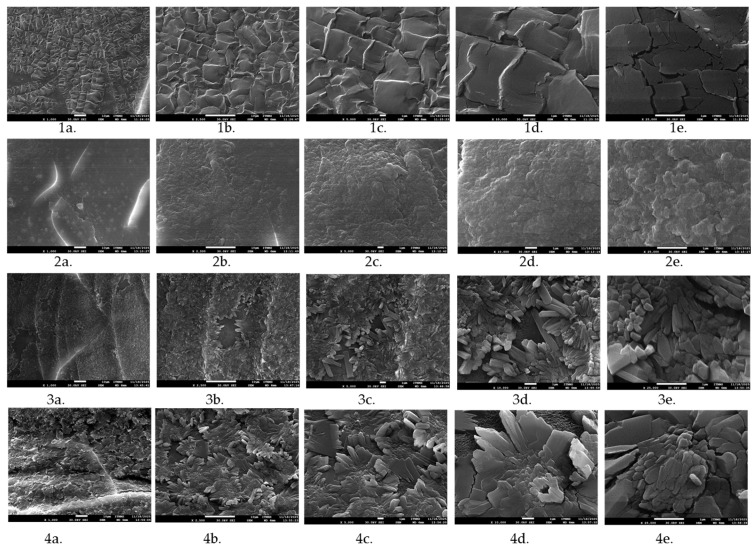
Morphology of neat Pull-MA hydrogel (**1a**,**1b**,**1c**,**1d**,**1e**), and microbiological suspension-loaded hydrogels: T3 (**2a**,**2b**,**2c**,**2d**,**2e**), T2 (**3a**,**3b**,**3c**,**3d**,**3e**), and T1 (**4a**,**4b**,**4c**,**4d**,**4e**) at different magnifications (2500; 5000; 10,000; 25,000×).

SEM images have demonstrated that neat xerogels exhibit more continuous structure in comparison to the microorganism suspension-loaded xerogels (Figure 5). As the amount of loaded suspension increases, the disruption of the structure becomes more apparent. The resulting gels are notable brittle, as evidenced by the fracture micrographs. The degree of surface irregularities on the surface of the granules was directly related to the proportion of cultivation broth.

### 3.4. Results of Swelling Properties Analysis

**Figure 6:**
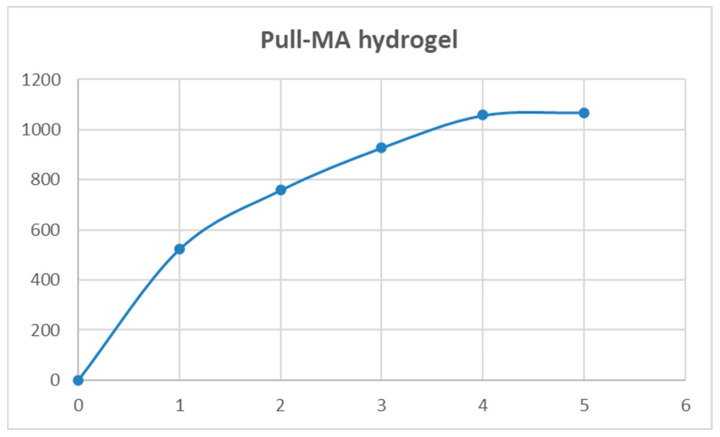
Swelling curve for Pull-MA hydrogel at pH 7 and room temperature.

The Pull-MA hydrogel reached an equilibrium swelling ratio (ESR) of 1068% within 5 h, indicating its suitability for broth delivery under these conditions, as shown in Figure 6. Although its ESR is 1.5–2.7 times lower compared to synthetic poly(N-isopropylacrylamide) and poly (N-isopropylacrylamide)/poly(acrylic acid) hydrogels, the Pull-MA system exhibits a more sustained swelling ability, maintaining water uptake for up to 12 h [31]. Importantly, unlike these synthetic counterparts, which are non-biodegradable, the pullulan-based hydrogel is derived from a renewable polysaccharide and is fully biodegradable, offering a more environmentally responsible alternative without compromising functionality.

### 3.5. Results of Gel Fraction Determination

After three measurements, the gel fraction value was estimated at 86.4% for neat xerogel, while the values for the loaded xerogels were 85.3%, 84.7%, and 84.6%, indicating that in situ incorporation of suspension does not affect crosslinking density. In other words, free-radical polymerization proceeds smoothly in the presence of the culture fluid, allowing the successful entrapment of cells and nutrients within the gel pores.

### 3.6. Results of the Seed Germination Test

Seed germination assays were conducted to evaluate the biological performance of the fertilizer-loaded Pull-MA hydrogels. Two model crop species, maize and pepper, were selected due to their agronomic relevance and distinct seed morphology, which enables assessment of hydrogel performance across different seed types.

**Figure 7:**
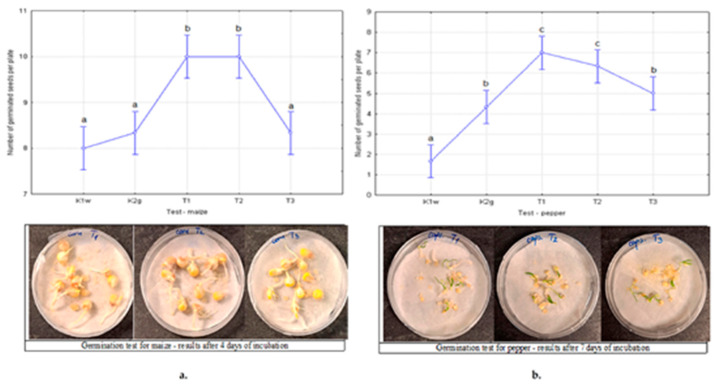
Seed germination after 4 days of incubation for maize (**a**) and 7 days of incubation for pepper (**b**). The figures show the mean values of the number of germinated seeds and the significance levels according to the applied Duncan multiple range test (marked with a lowercase letter). Treatments marked with the same letter are not significantly different at the 0.05 level.

The germination results for maize and pepper seeds for the control sample, neat hydrogel, and fertilizer–embedded hydrogel samples are presented in Figure 7a,b. Statistical analysis of the number of germinated maize seeds revealed a statistically significant difference between the treatments (*p* = 0.000064). This result further allowed for post-hoc analysis to determine the significance levels between groups (Figure 7a). At the same time, it was found that there was also a statistically significant difference in the treatments applied to peppers (*p* = 0.000011) (Figure 7b).

The results presented in Figure 7a show that there was no statistically significant difference between the controls K1w and K2g with the T3 treatment. For these treatments, the obtained SG values are 80%, 83.33% and 86.67%, respectively. On the other hand, treatments T1 and T2 are at a higher level of statistical significance with SG values of 100%. The result shows that with these treatments, all analyzed seeds germinated in 4 days. According to the results, the RSG values obtained for T1, T2, and T3 treatments are 1.08, 1.25, and 1.25, respectively. Therefore, it was expected that treatments T1 and T2 would have significantly higher relative seed germination values than treatment T3.

The germination results for pepper (Figure 7b) are very similar. Namely, the lowest values of germinated seeds appeared in the water treatment, K1w, with an SG value of 17%. Control 2, K2g, showed slightly better properties in pepper than in maize and was found at a slightly higher level of significance (lowercase b). What is similar in the test with maize and pepper is the significance shown by the T3 treatment, which can be classified as the control level K2g. Their SG values are 50% and 43%, respectively. Also, treatments T1 and T2 stood out as the best on peppers (lowercase c), with SG values of 70% and 63%, respectively. The results show that germination in pepper is significantly lower than in maize. The reason for this may be the pepper variety used in this study, which may have a lower germination rate.

Therefore, the T1 and T2 formulations showed a statistically significant increase (*p* < 0.05) in RSG, RRG, and GI compared to both the control and the T3 treatment, indicating enhanced seed vigor and early seedling establishment. The improvement can be attributed to the high swelling capacity of Pull-MA hydrogel, which maintained moisture availability in the immediate vicinity of the seed and created a favorable microenvironment for oxygen diffusion and nutrient uptake. Furthermore, the in situ incorporation of microbial inoculant likely contributed to faster metabolic activation and root elongation. These findings are consistent with previous studies, which demonstrate that biopolymer-based hydrogels and biofertilizer carriers significantly improve germination percentage and seedling vigor. For example, Rabaa et al. [32] reported that maize seeds treated with biopolymer-based inoculants under water stress exhibited higher germination and growth indices. Similarly, Prabhpreet et al. [33] emphasized that biopolymer coatings can mitigate abiotic stress by maintaining water balance during seed imbibition. At the same time, it is essential to note that the role of Trichoderma species as growth promoters has been extensively studied. This genus has great potential for use as a biofertilizer, which has been tested on maize, pepper, and other plants [34,35,36,37]. Garg et al. [38] observed that biopolymer-seed coatings enhanced uniform germination and stress tolerance in several crop species. Moreover, recent research on pullulan-based hydrogels has confirmed their potential as biodegradable, water-retentive matrices for seed coating and controlled nutrient delivery [39].

The presented results clearly demonstrate a gradual decline in the survival rate of microorganisms over time, with statistically significant differences observed between the time points. In the initial period (0–4 h), the survival rate remains high (77–83%), indicating that the process of incorporation into the hydrogel did not induce substantial initial stress. Similar observations were reported by John et al. (2011) [40], who showed that encapsulation within polymeric matrices is a relatively mild technique that ensures high initial cell viability.

However, from 12 h, a more pronounced decline in viability is observed, with values of ~68% (12 h) and ~61% (24 h), suggesting that the microorganisms within the matrix may be experiencing nutrient limitations, diffusion barriers, or mild osmotic stress. Similar behavior was described by Bashan et al. (2002) [41], who reported that microencapsulated microorganisms often exhibit a stable initial phase followed by a gradual decrease as available resources become depleted and the microenvironment within the matrix changes. After 48 h and 96 h, a further decrease is observed (53% and 51%), which is expected given that the survival of inoculants in carriers, particularly hydrogels and biopolymers, may decline by 30–60% within the first few days due to the gradual loss of metabolic activity and matrix aging.

**Figure 8:**
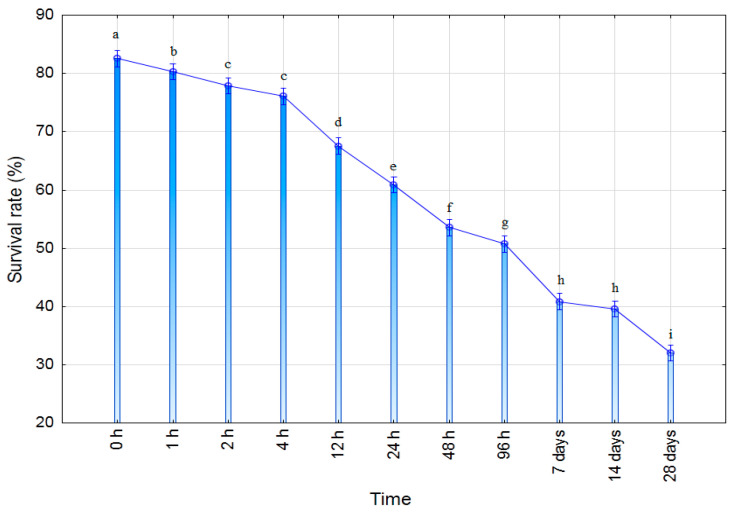
Viability test of *Trichoderma*-Pull-MA hydrogel in PBS solution (T2 hydrogel). Values followed by the same letter are not statistically significantly different at the 0.05 level (Duncan’s test) (n = 3).

In the period from 7 to 28 days, the survival rate decreases further, ranging from approximately 40% (7 and 14 days) to about 32% (28 days). This finding is consistent with the work of Schoebitz et al. (2013) [42], who reported that microorganisms encapsulated in bio-matrices can remain viable for more than a month, although with a substantial reduction in viability, most commonly to 20–40%, which is a range similar to that shown in Figure 8.

In comparison with other studies that utilized hydrogel matrices for microorganism immobilization, the Pull-MA hydrogel exhibits relatively stable Trichoderma survival up to 7 days and a moderate decline after 14–28 days, which can be considered favorable in the context of biofertilizer applications, where a viable population of >30% over several weeks is often sufficient to ensure effective colonization potential upon soil application [29,43]. Thus, the temporal dynamics indicate that the hydrogel is a suitable carrier for short- and medium-term retention of microorganisms, which is crucial for practical applications in biocontrol and biofertilization.

The superior performance of T1 and T2 likely arises from an optimal balance of release, microbial viability preservation, and moisture buffering at the seed surface [44,45]. Tests T1 and T2 provide sufficient initial availability of soluble nutrients and bioactive metabolites without inducing osmotic stress or burst release that would shorten the effective delivery [46,47]. In contrast, T3 may cause excessive local concentrations or reduced long-term viability of the inoculum, both undermining early seedling stimulation [48,49].

By analyzing the results obtained for root length, it can be concluded that the RRG values are highest in treatment T2 (1.34) and lowest in treatment T3 (1.08), and at the same time, the GI values are highest in treatment T2 (1.67) and lowest in treatment T3 (1.16). In accordance with the above, we can conclude that treatment T2 is the most suitable for maize treatment. Similar results were obtained for pepper. The lowest RRG and GI values were obtained for treatment T3 (1.25 and 3.75, respectively), while the highest RRG and GI values were obtained for treatment T2 (4.30 and 16.34, respectively). These results confirm the previously obtained results for maize, that treatment T2 showed the best growth characteristics for the applied seeds. The reason probably lies in the fact that the T2 sample has achieved a compromise between the amount of fertilizer and the release of *T. harzianum*.

The superior performance of the T1 and T2 (especially T2) formulations, therefore, reflects the synergistic effect of moisture retention, gradual nutrient release, and microbial activity, which is in good agreement with the results reported for other biopolymer-based seed coating systems. These results further confirm that Pull-MA hydrogels are promising candidates for improving seed germination efficiency and early seedling growth under different growing conditions, as the combination of adequate biofertilizer loading and appropriate release is crucial for promoting early stages of plant development.

## 4. Conclusions

The obtained results clearly demonstrate that pullulan methacrylate successfully incorporated functional groups into the Pull skeleton, as confirmed by FTIR and DSC analyses. The addition of methacrylate functional groups made it possible to prepare a hydrogel via free-radical polymerization at room temperature. The synthesized Pull-MA hydrogel exhibited a strong swelling capacity and quickly reached equilibrium, indicating its potential as an effective system for controlled nutrient delivery in agriculture. Biological germination tests further confirmed the hydrogel’s functionality. Incorporating microbial inoculant into the hydrogel matrix positively influenced germination, with treatment T2 (biopolymer: cultivation broth 1:5 *w*/*w*) being the most effective. This treatment maintained an optimal balance between nutrient concentration and release rate, resulting in the highest values of RSG, RRG, and GI for both tested seed types—maize and pepper. Conversely, treatment T3 (biopolymer: cultivation broth 1:7.5 *w*/*w*) showed efficiency similar to control samples, likely due to limited nutrient availability or slower release, particularly in pepper, which generally has lower germination potential. In conclusion, the Pull-MA hydrogel, especially in the T2 formulation, shows strong promise as an advanced matrix for controlled nutrient delivery, promoting better germination and early plant growth. These findings support the application of these Pull-MA hydrogels as carriers for microbial fertilizer and provide a solid foundation for further system optimization and validation under real field conditions. However, considerations of process scalability, economic feasibility, and field application challenges remain critical for practical implementation. The hydrogel’s simple room-temperature synthesis and the commercial availability of pullulan suggest potential for cost-effective large-scale production. Further studies are required to evaluate its performance under field conditions, including interactions with native soil microbiota and varying climatic factors, to ensure effective microbial viability and biofertilization

## Data Availability

The original contributions presented in this study are included in the article. Further inquiries can be directed to the corresponding author.

## Abbreviations

The following abbreviations are used in this manuscript:

GMA	Glycidyl methacrylate
MBAM	N, N′-methylenebisacrylamide
TEMED	*N, N, N′, N′*-tetramethylethylenediamine
DMSO	Dimethyl sulfoxide
PPS	Potassium persulfate
DMAP	4-dimethylaminopiridine
ESR	Equilibrium swelling ratio
RSG	Relative seed germination
SG	Seed germination percentage
GI	Germination index
FTIR	Fourier-transform infrared spectroscopy
DSC	Differential Scanning Calorimetry
